# 5HTTLPR Genetic Variant and Major Depressive Disorder: A Review

**DOI:** 10.3390/genes11111260

**Published:** 2020-10-26

**Authors:** Caroline Fratelli, Jhon Siqueira, Calliandra Silva, Eduardo Ferreira, Izabel Silva

**Affiliations:** 1 Postgraduate Program in Health Sciences and Technologies, Campus Faculty of Ceilandia, University of Brasilia, Brasilia 72220-275, Brazil; carolfratelli@gmail.com; 2 Department of Pharmacy, Campus Faculty of Ceilandia, University of Brasilia, Brasilia 72220-275, Brazil; jwillatan@gmail.com (J.S.); cdssilva@gmail.com (C.S.); eduardoantonioferreira@gmail.com (E.F.)

**Keywords:** genetic polymorphism, 5HTTLPR, risk-factors, nervous system, pharmacogenomics, major depressive disorder

## Abstract

Major Depressive Disorder (MDD) is a disease that involves biological, psychological, and social interactions. Studies have shown the importance of genetics contribution to MDD development. The SCL6A4 protein (5HTTLPR) functions transporting serotonin, a neurotransmitter linked to mood and emotion, to the synaptic cleft. Hence, this study seeks, through a literature review, a better comprehension of the 5HTTLPR genetic variant association with MDD. For this purpose, a search was performed on the Virtual Health Library Portal for articles that related 5HTTLPR to MDD. Most of the articles found were conducted in the American continent, with one (1) study implemented in Brazil. 5HTTLPR associations were found regarding changes in the nervous system, pharmacology, and risk factors seen in MDD patients. When verifying the allelic distribution, the S allele had a higher frequency in most of the studies analyzed. Despite not finding a commonality in the different studies, the tremendous genetic variation found demonstrates the MDD complexity. For this reason, further studies in diverse populations should be conducted to assist in the understanding and treatment of the disease.

## 1. Introduction

Characterized by sadness, loss of interest or pleasure, feelings of guilt or low self-esteem, altered sleep and appetite, tiredness or lack of concentration, depression is a common mental disorder and one of the foremost causes of disability worldwide [1,2,3,4]. It tends to affect more women than men and may lead to suicide [1].

The causes of Major Depressive Disorder (MDD) are compound and far from being understood, as it involves biological, psychological, and social interactions. The genetic contribution to MDD is approximately 35%, with heredity having the highest percentage. These findings suggest that different genetic variables may contribute to the risk of developing MDD [5,6,7,8].

The 5-hydroxytryptamine transporter (5HTT), encoded by SLC6A4 (solute carrier family 6, member 4), also known as sodium-dependent serotonin transporter, is found in the plasmatic membrane and transports the neurotransmitter serotonin from the synaptic cleft into presynaptic neurons for recycling, thus terminating its action [9,10]. Hence, 5HTT is involved in the serotonergic neurotransmission regulation by mediating the serotonin availability to other serotonergic systems [9,10].

5HTTLPR-VNTR is one of the described genetic variants of the SLC6A4 gene. Located on human chromosome 17q11.2, this polymorphism is biallelic with a 44 bp insertion/deletion flanking the 5′ promoter region of the gene that gives rise to two different alleles: a long allele (Long, L), which contain the insertion, and a short allele (Short, S), which contain the deletion [9,11,12]. Population genetic studies have shown that the S allele presence decreases the 5HTT promoter gene’s transcriptional efficiency, resulting in a lower serotonin transporter binding and its uptake [13]. Accordingly, this genetic alteration may increase the risk of susceptibility to psychiatric disorders, such as MDD [13].

Along with the extensively studied 5HTTLPR-VNTR, the single nucleotide polymorphisms (SNP) rs25531 and rs25532, which refer to the same nucleotide located within the promoter region (5HTTLPR), implements a new variation into the allelic frequency and contributes to the alteration of the protein functionality [14]. The SNP rs25531 can modify the S allele and the L allele of the 5HTTLPR-VNTR variant leading to the appearance of allelic subtypes: Sa, Sl, La, Lg. The substitution of the nucleotide base (A/G) influences the transcriptional regulator AP2 (activation protein 2) functionality that normally acts as an activator or repressor of transcription. In the S, La, and Lg alleles, this SNP creates an AP2 binding site that exerts a repressive role on the SLC6A4 promoter activity, hence suppressing SLC6A4 transcription [14,15,16].

Thus, this study seeks, through a literature review, a better comprehension of the 5HTTLPR (VNTR or rs25531) genetic variant association with Major Depressive Disorder.

## 2. Materials and Methods

### 2.1. Search Strategy

The research was performed on 26 March 2020, through the Virtual Health Library (VHL) Portal for studies that referred to the 5HTTLPR genetic variant and Major Depressive Disorder (MDD) association. For this, the following descriptors were used: 5HTTLPR polymorphism AND major depressive disorder.

The findings were selected by reviewers, ensuring that all were within the established criteria.

### 2.2. Filters, Inclusion, and Exclusion Criteria

The selected articles must have been published between 2016 and 2020, in the languages English or Spanish.

The inclusion criteria were: (1) studies should relate in some way to a genetic variant of 5HTTLPR and MDD; (2) present the laboratory methods used; (3) be original articles.

Articles were excluded if they: (1) presented incomplete data, including statistical data; (2) was not focused just on MDD; (3) were systematic reviews or meta-analysis; (4) were duplicated.

### 2.3. Data Extraction

Data were extracted from articles in accordance with pre-established criteria. For each study, the following data were obtained: author, study title, objective, year of publication, the country in which the study was conducted, type of genetic variant (SNP or VNTR), sample size, laboratory methodology, main result, *p*-value, and genotypic frequency.

## 3. Results

**Figure 1:**
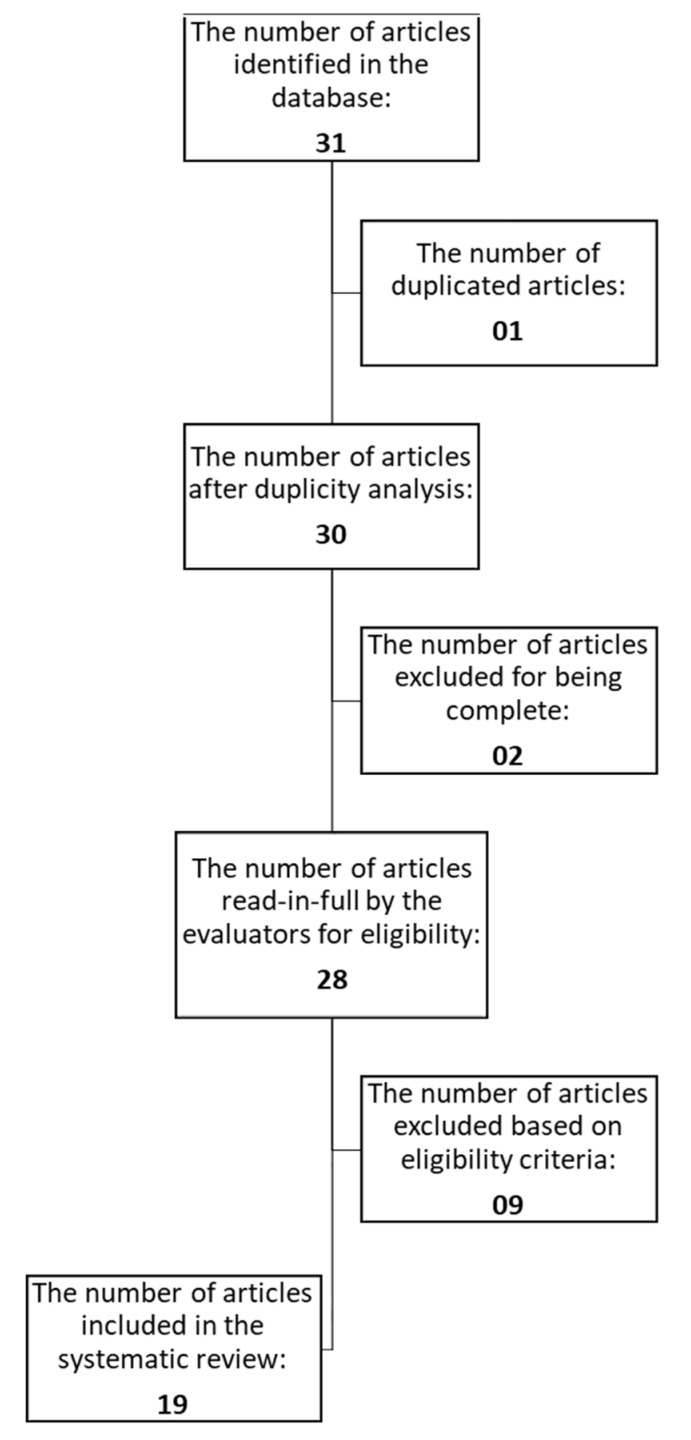
Article selection flowchart.

In summary, the literature search resulted in 31 articles, and after the application of the established inclusion and exclusion criteria, a total of 19 original articles remained, as described in Figure 1.

**Figure 2:**
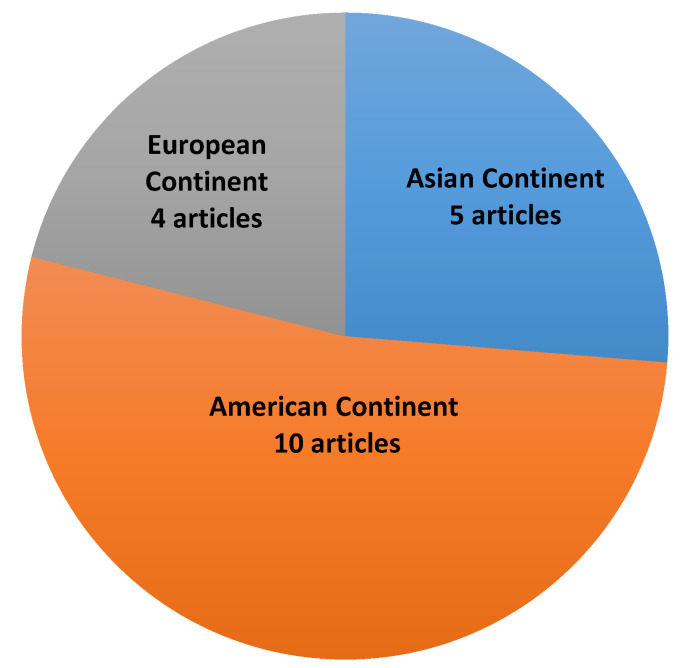
Number of articles per continent.

As shown in Figure 2, most of the selected articles were from the American continent, followed by the Asian and European continents. Only one Brazilian article was found; this study was conducted in Minas Gerais and published in 2018 (Table 1).

## 4. Discussion

### 4.1. HTTLPR Variant Genotypic Distribution in the Population

**Figure 3:**
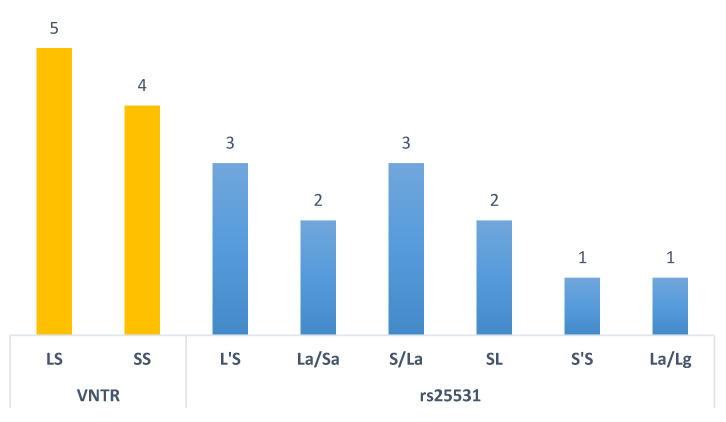
Genotypic distribution according to the number of articles found.

Genetic variants may change according to the studied population. Accordingly, when analyzing the 5HTTLPR-VNTR variant in the selected articles, the S allele had a higher genotypic frequency. Whereas in the 5HTTLPR-rs25531 variant, the heterozygous LS genotype was the most frequent (Figure 3). As the 5HTTLPR-rs25531 variant can be present in the 5HTTLPR-VNTR variant, the authors grouped the variants to facilitate comprehension. According to Tatham et al. [40], the L’L’ group represents LA/LA; L’S’ represents a long allele and an efficient transcription (La/Lg or La/Sa) allele; and S’S’ represents two alleles with low transcriptional efficiency (Sa/Sa; Lg/Sa or Lg/Lg). While, Kao et al. [27] classified the variants as: S’S’ (Lg/Lg + Lg/S + S/S); L’S’ (La/Lg + Lg/S); L’L’ (La/La). SL will be for 5 HTTLPR-rs25531 and LS for 5HTTLPR-VNTR to facilitate the readers’ understanding.

In general, the S allele is present in 42% of Caucasians and 79% of Asians, while the L allele is much more frequent in Western populations than in Asian populations [14]. A Caucasian population study with 46 MDD patients and ages between 19 to 58 years has shown that the S allele of the 5HTTLPR-VNTR variant may be related to the increased risk of depression by negatively affecting the serotonin reuptake rate due to its low expression [39]. Mendonça et al. [19], in a study developed in Brazil, had the S allele as the most frequent in their sample, a result similar to Murdoch et al. [41] study in an Asian population, in which more than 60% of the MMD samples had the present allele. Hence, the S allele may be related to the depressive risk, plausibly due to its adverse effects on the serotonin reuptake rate [14]. Nevertheless, Kao et al. [27] found no correlation between the S allele and the risk of developing depression.

According to Sun et al. [35], the combination of the short and long allele of 5HTTLPR-VNTR in heterozygous individuals has a statistically higher chance of MDD development (OR = 1.42, *p* = 0.02) than those with homozygous genotype. Similar results were found in Turkish [21], Mexican [20], and Indian [36] populations.

On the other hand, the 5HTTLPR-rs25531 variant expression is highly diversified among the distinct populations, as different genotypic frequencies may be detected within the same geographic region. These differing genotypic frequencies have been observed in studies in the North American population (Canada, United States of America, and Mexico) that demonstrated a variation in the SL genotypic domain [30,33], though this might also be due to the ethnicity variation in these regions.

The 5HTTLPR-rs25531 S/La genotype was the most prominent in a study by Camarena et al. [18] with Mexican individuals (53 research participants). Jaworska et al. [34], in a study with a Canadian population, obtained an equivalent result but with a sample size of 20 participants. In two separate studies, Tatham et al. [39,40] found the L’S’ domain, representing one high and one low transcriptionally efficient alleles, in most of their studied population (La/Lg ou La/Sa). A comparable result was attained by Ramasubb et al. [38], in which the La/Sa genotype was also more frequent.

In Asia, a variety of genetic domains are seen within the same population. Basu et al. [17] found the 5HTTLPR-rs25531 SL genotype in MDD individuals from India. Whereas Manoharan et al. [36] found a higher frequency of 5HTTLPR-rs25531 S/La genotype in the Indian population. Nonetheless, in a study with Chinese individuals, most research participants had the 5HTTLPR-rs25531 S’S’ genotype (Lg/Lg + Lg/S + SS). This S’S’ genotype, according to Kao et al. [27], had not been found in previous studies and was classified as a new genotypic domain.

European studies are similar to the others, as they also found a higher frequency of the SL genotype [32]. By analyzing Dutch individuals’ genotypes, Fleurkens et al. [22] noted that 52% (*n* = 109) of the research participants had the La/Lg genotype. In another German study by Schneider et al. [28], however, had the La/Sa genotype, a result similar to the study conducted in a Canadian population [39,40]. Thus, it can be inferred that these genotypes are found more frequently in those of European ancestry.

The tremendous genetic variability seen in these studies attests that the world population’s genetic dissimilarity. Hence, research into allelic variations in different populations’ genetic background is critical in understanding the existing diseases and assisting in their prevention and treatment in each society.

### 4.2. HTTLPR Variant and the Nervous System

The nervous system is responsible for coordinating actions in the human body through chemical (neurotransmitter) and electrical signals between its cells. Among the more than fifty neurotransmitters described, serotonin (5-HT) stands out, as it is responsible for regulating sleep and mood [42,43].

According to Han et al. [23], L allele carriers, in the 5HTTLPR-VNTR variant, obtained a significant reduction in the right anterior midcingulate gyrus (*p* = 0.001) and the cortical (*p* = 0.001) volume, in comparison to S allele homozygous individuals. Through a surgical cut of both lateral bundles of this rotation, it is possible to interrupt the neural Papez circuit communication and reduce the level of pre-existing nervous depression and anxiety; it can also aid with obsessive-compulsive disorder (OCD) treatment, chemical addiction, and chronic pain [44]. Therefore, this volume reduction in the patient with the L allele is considered a “protective factor” [23]. In contrast, the SS homozygous depressed patients’ hippocampal volumes were smaller than healthy controls in both hemispheres [43]. The effects of the 5HTTLPR on hippocampal volumes were also detected in OCD patients [45].

Ancelin et al. [46] demonstrated that a lifetime of MDD was associated with persistent volume reductions in the deep nuclei, insular, thalamus, ventral diencephalon, pallidum, and nucleus accumbens and with a broader pericalcarine region in both men and women, and other gender and age-related changes. In regards to the 5HTTLPR genotype, Ancelin et al. [46] study found no significant volumetric differences according to 5HTTLPR within the groups with or without lifetime MDD but did find that the lifetime MDD and LL genotypes participants had smaller thalamus while the lifetime MDD and SL genotypes participants had larger pericalcarine and lingual volumes compared with their non-MDD counterparts. Though these results should be taken into careful consideration as in younger populations, the S allele is perceived as a risk factor for mental and physical distress, whereas in older adults, the LL genotype appears to be a risk factor for those highly exposed to chronic disorders and severe stressors [47].

Tatham et al. [40], in separate studies, evaluated the integrity of the brain white matter and reported increased lesions in 5HTTLPR-VNTR S’L’ heterozygous patients when compared to SS and LL homozygous. Furthermore, the interaction between fractional anisotropy in the uncinated fascicle on the right and 5HTTLPR altered the percentage in the severity of depression [40]. Additionally, the putamen and left thalamus region in the La/La homozygotes of the 5HTTLPR-rs25531 variant had a higher volume than the SS homozygotes and La/S heterozygotes, which could negatively related to behavior, complex and sequential motor planning, learning, cognitive, and motivational direction, and in some cases surgery is needed [34].

Monoamine serotonin’s binding level to the 5HTT receptor is related to emotional processing, where a lower serotonin transporter binding is associated with psychiatric symptoms [30,48,49,50]. In a study coordinated by Schneck et al. [30], a higher reactivity of the right amygdala was associated with lower binding to the 5HTT raphe nucleus, but not to the 5HTTLPR genotype. Research on *SLC6A4* AluJb methylation in MDD and amygdala reactivity, in addition to its associations with 5HTTLPR-rs25531 and stress, in depressed patients revealed that individuals with low methylation, in conjunction with a shorter MDD history and lower amygdala reactivity, may present an epigenetic process more adaptable to stress [13,14,28,51]. *SLC6A4* hypermethylation has typically been described as independently associated with early stress and depressive disorders; though, very few studies address whether methylation can mediate the interaction between stress and 5HTTLPR in predicting psychopathological risk [13]. When assessing the association of raphe nuclei abnormal echogenicity in MDD patients’ brainstem, Kotisc et al. [32] detected a frequency of 66% of abnormality in MDD individuals.

In synthesis, the 5HTTLPR genetic variant, be it VNTR or rs25531, has some role in the Nervous System in MDD patients.

### 4.3. HTTLPR Variant and MDD Risk Factors

Major Depressive Disorder (MDD) is a disease with a complex etiology that depends on diverse genetic and environmental factors for its development. 5HTTLPR variant studies with biological and cognitive factors underlying depression, such as childhood trauma, attempted suicide, stress, and anxiety, have been reported in different populations, thus contributing to a better understanding of the disease. The S allele of 5HTTLPR increases the risk of depression only in stressed individuals [52,53]; therefore, it should not be widely generalizable, only observable in limited situations and modest sample size [52,54,55]. On the other hand, the L allele’s increased transcriptional activity is considered protective against depression, yet it has been associated with suicide, nicotine dependence, and attention deficit hyperactivity disorder [54,55].

In order to verify facial expressions in Dutch depressive patients in a context of 5HTTLPR-rs25531 gene polymorphism and childhood trauma, Fleurkens et al. [22] found that more than 60% of the studied sample had no trauma in childhood, which may be seen as a confounding factor since the information was obtained by self-report. Although not finding a genotypic association of the present gene with childhood adversity (*p* = 0.128), depressed S/Lg genotype patients with childhood trauma, compared to those with the La/La genotype, avoided sad facial expressions, even after having their depressive and anxiety symptoms controlled. Furthermore, the S/Lg heterozygous group had less emotional and psychological resilience than the La/La homozygous group; that is, they were less able to deal with their problems, overcome obstacles and resist pressure, whether emotional or psychological [22].

Similar to Fleurkens et al. [22], other studies have failed to find a statistical association between the 5HTTLPR polymorphism and childhood trauma [21,40,56,57]. In a Canadian study with the 5HTTLPR-VNTR variant, of 55 MDD carriers analyzed, the SS genotype was not significantly associated with childhood trauma when compared to those of the LL genotype (*p* > 0.05) [40]. Özçürümez et al. [21] also did not find an interaction between childhood trauma and the 5HTTLPR-VNTR variant in MDD patients residing in Turkey (*p* = 0.28). Despite not detecting this interaction, S allele carriers showed a higher risk of developing depressive symptoms in response to adversity in childhood than individuals with the L allele, which corroborates with similar results found in the literature [53,58]. Moreover, they believe that the risk of developing depression depends on the amount of adversity suffered in childhood. This hypothesis agrees with epidemiological studies [59] that states that people who suffer abuse in childhood are twice as likely to develop depression [21].

Despite these findings, it is essential to highlight the authors’ significant limitation: the reliability in the participants’ memory [21,22]. Depressed adults tend to remember more the negative points experienced in childhood than the positive ones, which may influence the studies’ results. Nonetheless, according to Özçürümez et al. [21], the consideration of childhood mistreatment history may lead to a richer comprehension of the clinical differences, genetic foundations, biological correlates, and studies’ results linked to MDD.

Suicide is another MDD associated comorbidity that generates a considerable public health concern. To determine the association of the 5HTTLPR-VNTR variant with the suicide attempt and its comorbid disorders, Sarmiento-Hernández et al. [20] researched 200 Mexican adolescents (11 to 18 years of age) with depression and that had attempted suicide in the last six months prior to the survey. Their most commonly used suicide techniques were nonviolent methods, such as drug overdose [60,61,62]; however, the participants also described cutting and hanging [20].

Analyzing the genetic aspect of the Sarmiento-Hernández et al. [20] study, the results support the association between the S allele, or the SS genotype, and suicide. Patients with low-expression 5HTTLPR genotypes and childhood trauma have an increased risk of suicidal behavior [63]. Some studies support an increased risk of suicide attempts in depressed patients with the S allele [64,65,66], though other studies have not found this association [67,68,69,70,71]. Sarmiento-Hernández et al. [20] conclude that the higher S allele frequency in the studied population reinforces the hypothesis that the 5HTTLPR variants play an essential role in the development of suicidal behavior in depressed adolescents, regardless of the presence of another psychiatric comorbidity [20].

Depression is a disease with a higher hereditary prevalence within families’ lines than the families without a member with depression [29]. The presence of the S allele as a risk factor was statistically associated with depression in a study that analyzed the 5HTTLPR-VNTR variant in Turkey (*p* < 0.001), in which more than 70% of depressed patients had the S allele [21].

In a 30-year longitudinal study of Caucasian biological descendants conducted in the United States, the participants had moderate to severe depression with no history of life adversity or any other psychiatric problem reported in the eight years that preceded the research [72,73]. Bansal et al. [29] sought to assess whether the 5HTTLPR-VNTR variant modulates the heritable risk and the S allele frequency between the high heritable risk (HHR) and low heritable risk (LHR) groups was not statistically significant (*p* = 0.3390). Analyzing the cerebral cortex, the LHR group had the S allele associated with a thinning of the cortex, unlike the HHR group, which had the S allele associated with a thickening of the cortex. Therefore, the 5HTTLPR-VNTR variant may accentuate the heritable depressive risk effects on the cortex by probably modulating brain plasticity [29].

Talati et al. [33], in an epigenetic study carried out in the United States with 203 research participants, demonstrated that 64% of the studied family members had a high risk for depression. These high-risk participants had two copies of the S allele in the 5HTTLPR-rs2553 polymorphism, as well as: higher impulsiveness (*p* = 0.0013), hostility (*p* = 0.017), and neuroticism (*p* = 0.013). In other words, the people who acted hastily, without thinking or analyzing the situation, are contrary and estranged, and tend to experience bad occurrences in normal life situations (envy, anger), had a high frequency of the SS genotype. Furthermore, these participants noticed higher fear-based anxiety disorder rates, but not in the other diagnoses [13,14,51].

Although a Brazilian study, conducted by Mendonça et al. [19], showed that approximately 70% of the studied children (*n* = 40) that lived with depressed mothers (*n* = 40) exhibited some form of psychiatric disorder, be it depression, generalized anxiety, or Attention Deficit Hyperactivity Disorder (ADHD). Moreover, of the 40 depressed mothers studied, half had a family history of depression. In a study investigating the association of the 5HTTLPR polymorphism and the CpG (5mC) DNA methylation levels of the AluJb repeat element in the *SLC6A4* promoter region (5HTTLPR) of a mother–child exposed to maternal depression, no correlation was found between the S allele and the pattern of depression occurrence between mother and child (*p* < 0.999), even though this association was seen in other studies [19,33,74].

The high S allele frequency found in the Mendonça et al. [19] study is not common in the Brazilian population [41], in which more than 60% of the sample had the S allele. Consequently, the authors believe that, although the high frequency may be a risk factor for developing depression in the Brazilian population, it is not sufficient to cause depression in groups of depressed mothers and children or just depressed mothers [19].

Regarding DNA methylation, depressed mothers and children had a reduction in methylation levels. This decrease was more pronounced when only the S allele carriers were analyzed. Consequently, as a hypothesis, lower levels of AluJb methylation may be considered an epigenetic marker for depression in children exposed to maternal depression [19]. However, this hypothesis should be analyzed only as part of the molecular context that contributes to the diagnosis of childhood depression since other factors not characterized in the study may contribute to the AluJb methylation [13,19].

Thus, in addition to AluJb methylation being considered a possible potent epigenetic marker for depression in children from depressed mothers [19], a German study has shown a possible association of AluJb methylation with recent stress in 122 MDD research participants [28]. When verifying the AluJb methylation in the 5HTTLPR-rs25531 gene and recent stress association, Schneider et al. [28] found an interaction of gene versus environment when associated with methylation. Confronted with recent stressful experiences, carriers of the risk allele (S/Lg) showed lower AluJb methylation compared to LaLa homozygotes (*p* = 0.003). Furthermore, Yeh et al. [75] suggest that lower AluJb methylation may inhibit 5HTTLPR gene expression.

### 4.4. HTTLPR Variant and Pharmacotherapy

The therapeutic response can be seen as an aggregated factor, with genetics as one of its variables. Experimental evidence has demonstrated the relationship between the 5HTTLPR genetic variant and the pharmacotherapy used by MDD patients [27,36]. The strong relationship of the L allele with a selective serotonin reuptake inhibitor (SSRI) has been seen in studies of different populations [18,36,38,76], as well as the S allele relationship with SSRIs [25,77,78,79,80].

In a study by Manoharan et al. [36], the LL genotype of the 5HTTLPR-VNTR variant demonstrated a strong association with the response to fluoxetine in MDD patients in South India (*p* = 0.0066). Carriers of this genotype reduced the score on the Hamilton Depression Rating Scale (HAM-D) when compared to genotypes that had at least one S allele, confirming that S allele carriers are less likely to respond to the drug. The same finding was detected in Indian [81], Chinese [82], and Caucasian studies [83,84,85], in which the LL genotype provides a better response to SSRI treatment.

Notwithstanding, in a Thai study, Kao et al. [27] observed a significant association between the gene polymorphism and the pharmacological treatment (reduction of the HAM-D score). Depressive participants with the LL genotype (*p* = 0.042) had a weak response to treatment with Duloxetine or Paroxetine, both SSRIs, i.e., there was no reduction in the HAM-D score. At the same time, Asian studies have shown a high S allele frequency in patients who have had a good therapeutic response [18]. In the Caucasian study conducted by Tatham et al. [39], there was a positive response in the association of pharmacological treatment with the reduction of the HAM-D score (*p* < 0.0001), but it was not related to the genetic variant.

Ivanets et al. [78], in a Russian study, sought to verify the influence of the 5HTTLPR-VNTR variant in determining depressive disorder remission levels and the risk of developing side effects during SSRIs use. In this study, SS genotype participants had an inadequate response to the treatment compared to the other genotypes of this variant (*p* = 0.11), their MDD remission level was significantly worse (*p* = 0.05), and developed the most severe side effects (*p* = 0.02) [78,80]. In another study [77], the S allele presence in the 5HTTLPR-VNTR variant was associated with the low efficacy of antidepressants in depressed women compared to men and, although they sought to find answers to this difference in clinical and demographic characteristics, no data could explain the low drug efficacy. Thus, they believe that the genders’ drug efficacy difference is related to the 5HTTLPR-VNTR variant genotypes [25,77,79].

By assessing the correlation between Fluoxetine response and 5HTTLPR-rs25531 variant, Manoharan et al. [36] found no statistically significant association (*p* = 0.0818), though their sample size may justify this. The same rationale was used to explain the same lack of correlation in depressed Mexican patients [18].

In this Mexican study, when evaluating the HAM-D score in response to the drug administered, a high frequency in the low activity (S) alleles was observed in patients that did not respond to fluoxetine (*p* = 0.0102), but this may be related to low sample size. Moreover, as the therapeutic response has a complex phenotype, the difference in results may be due to this heterogeneity. Accordingly, if heterogeneity is reduced, it increases the possibility of identifying the genetic variants involved in the pharmacotherapeutic response. For this reason, multicenter studies should be taken into account [18].

It is interesting to note the gender difference regarding the therapeutic response, as women seemed to respond better to treatment than men [18]. This disparity may be due to biological factors, such as menopause, or merely the higher rate of adherence to treatment by women [18]. However, this finding must be verified in other studies, given that more than 70% of the studied sample was female, a percentage no different from other studies [27,36,39].

In addition to Fluoxetine, Paroxetine, and Duloxetine, Ramasubbu et al. [38] decided to study Quetiapine XR (atypical antipsychotic) and Citalopram (SSRI) in Caucasian MDD research participants to examine the impact of the two drugs, with a differentiated mechanism of action, on the serotonin transporter and evaluate their tonsil (amygdala) response per 5HTTLPR-rs25531 polymorphism in MDD patients. The two treatments resulted in brain changes of different patterns in patients with the S/Lg genotype, despite a similar clinical improvement level. These differential responses may reflect an interaction between the genotype and the pharmacological effects. S/Lg patients treated with Quetiapine appear to have suppressed responses in the amygdala, which mediated their antidepressant effects. Regarding Citalopram, 5HTTLPR may modify SSRI drugs’ effect in the tonsil response to emotions considered negative. Hence, it is assumed that amygdala suppression is sufficient for a positive response to MDD treatment [38].

#### Limitations and Recommendations

With the promise of a comprehensive human genome sequencing and the success of polymorphism and gene–disease association research, the interest in genetics in the health area has increased in the following years. Whether genetic or environmental, understanding risk factors may help direct diagnostics and interventions, preventive or therapeutic, in complex disorders like MDD. These risk factors complement each other, and to implement any prediction of a disease’s risk also requires a comprehensive assessment of genetic risk [86,87,88].

Thus, research on new genetic markers that may assess a disease’s risk in different populations appears more frequently in literature. Information, such as study design, variables definition, sample calculation, statistical methods, participants selection, is essential in any study and brings quality to the report. Therefore, insufficient information diminishes the quality of the research report. It precludes an accurate assessment of research strengths and weaknesses, making it difficult to replicate the study in other populations, a situation that is strongly recommended in gene association studies [86,89,90,91].

Guidelines that assist in the quality of a scientific project have been published for several research projects, such as Strengthening the Reporting of Observational Studies in Epidemiology (STROBE) and Genetic Risk Prediction Studies (GRIPS) [86,92]. In this context, our group applied the 22 GRIPS items to evaluate the 19 selected articles from Table 1. Of these, 89.5% of the articles did not comply with at least 6 of the 22 items—percentage adequacy below 75%. The primary non-compliance was the lack of a clear description of the sample size or the sampling strategy. However, 89% of the selected articles selected described their study’s limitations, including the sampling itself (see Table S1 in the supplementary material).

Regarding sampling, the sample size is vital as small sample size decreases the study’s power and limits generalizability; large sample sizes are similarly at a disadvantage because of the inherent heterogeneity due to population stratification. The selection of the study’s participants is likewise essential for choosing participants free of other psychiatric and medical comorbidities avoids confounders. 

## 5. Conclusions

Major Depressive Disorder (MDD) is a compound disease involving genetic and environmental factors. Several studies have been conducted in different populations aiming to comprehend the biological, environmental, pathophysiological, and pharmacogenomic mechanisms involved in the development of this disease that has a significant impact on public health. 

The 5HTTLPR genetic variants are involved with several aspects of this disorder; however, not all findings generate a universal agreement in the scientific community. Therefore, genetic studies with different populations associated with multiple environmental factors are recommended to contribute to the understanding and treatment of the disease and may provide MDD patients with a better quality of life.

## Figures and Tables

**Table 1:** Comparison of different studies relating to the 5HTTLPR genetic variant and Major Depressive Disorder (MDD).

Author	Title	Objective	Year	Country	Sample (N)	Genetic Variant	Laboratory Tests	Results	*p*-Value	Genotypic Frequency
Basu et al. [17]	A preliminary association study between serotonin transporter (5HTTLPR), receptor polymorphisms (5-HTR1A, 5-HTR2A) and depression symptom- clusters in a north Indian population suffering from Major Depressive Disorder (MDD)	Detect associations between any factorial structure and the serotonin transporter (5HTTLPR) and the receptor (5HTR1A, 5HTR2A) polymorphisms in the northern Indian population.	2019	India	80	VNTR	Sequencing and MALDI-TOF	The studied population had a frequency of 72% in the S allele and no LL genotype. Despite finding a significant association between the L allele and the “detachment” factor, definite conclusions cannot be drawn.	Polymorphism × Psychic Anxiety = 0.96	VNTR = LS
						SNP (rs6295)			Polymorphism × Detachment = 0.07	
						SNP (rs6311)			Polymorphism × vegetative function = 0.42	
						SNP (rs6313)			Polymorphism × Pessimistic humor = 0.31	
Camarena et al. [18]	Association Study Between Serotonin Transporter Gene and Fluoxetine Response in Mexican Patients with Major Depressive Disorder.	Analyze the clinical association between 5HTTLPR polymorphism and the response to fluoxetine in Mexican MDD patients.	2019	Mexico	150	SNP (rs25531)	PCR	There was an increased frequency of low activity (S, Lg) alleles in patients who did not respond to fluoxetine when compared to those who did. Nonetheless, there was no statistical difference in the allelic analyzes.	Genotypic = 0.165 Allelic = 0.0637	S/La
Mendonça et al. [19]	Epigenetic variation at the SLC6A4 gene promoter in mother–child pairs with major depressive disorder	Investigate the association of the 5HTTLPR polymorphism and the CpG (5mC) DNA methylation levels of the AluJb repeat element in the SLC6A4 promoter region of a mother and child exposed to maternal depression.	2019	Brazil	40	VNTR	PCR, sequencing and real-time	Most participants (mothers and children) had the SS genotype (53.4%). In the findings, approximately 70% of children living with depressed mothers exhibited a psychiatric disorder, such as depression, generalized anxiety disorder, and attention deficit hyperactivity disorder. Differences in methylation levels appear to be influenced by the S allele.	Polymorphism × Mother and child ≤ 0.999	SS
Sarmiento-Hernández et al. [20]	Association between 5HTTLPR polymorphism, suicide attempt and comorbidity in Mexican adolescents with major depressive disorder.	Determine the association of SLC6A4 gene polymorphic variants in Mexican adolescents with MDD and attempted suicide and its comorbid disorders.	2019	Mexico	200	VNTR	PCR	There was a statistical difference in allelic distribution between the case and control groups. The S allele (251) frequency, in the case group, is higher than the L allele (149). Moreover, the findings support the hypothesis that the increase in SS genotypic frequency contributes to suicidal behavior in people with depression.	Genotypic = 0.004 * Allelic = 0.0009 *	LS
Ozçurumez et al. [21]	No Interaction Between Childhood Maltreatment and Serotonin Transporter Gene in Recurrent Major Depressive Disorder: A Clinical Sample	Investigate the interaction between specific forms of childhood mistreatment and the 5HTTLPR polymorphism in recurrent MDD in a clinical sample.	2019	Turkey	70	VNTR	PCR	Most recurrent MDD participants had the SL genotype (47.1%); this was mainly seen in the case group (43.4%). There was no interaction between child abuse and the 5HTTLPR polymorphism in relation to recurrent MDD.	Polymorphism × Heredity ≤ 0.001 * Mistreatment × Polymorphism = 0.28	LS
Fleurkens et al. [22]	Automatic approach-avoidance tendencies as a candidate intermediate phenotype for depression: Associations with childhood trauma and the 5HTTLPR transporter polymorphism	Investigate the role of the automatic influence of approach-avoidance trends as an intermediate phenotype candidate for depression, in the context of genes (5HTTLPR polymorphism) and childhood trauma.	2018	Netherlands	209	SNP (rs25531)	PCR	The group with S/Lg genotype carriers and no childhood trauma had most of the research participants (94 individuals). S/Lg heterozygous patients (higher of risk of depression) and suffered childhood trauma avoid sad facial expressions more than those with the LaLa genotype and who suffered trauma. Additionally, the automatic approach-avoidance trends may be an intermediate candidate phenotype for depression.	Polymorphism × Adversity in childhood = 0.128	La/Lg
Han et al. [23]	The effects of 5HTTLPR and BDNF Val66Met polymorphisms on neurostructural changes in major depressive disorder	Investigate the effects of the 5HTTLPR and BDNF Val66Met genetic variants polymorphisms and their interactions with MDD on the cortical volume and white matter integrity.	2018	Republic of Korea	95	VNTR	Magnetic resonance and genotypic analyzes, according to the protocols of Han et al. [24]; Smits et al. [25]; Wang et al. [26]	Of the 95 MDD participants, the most (54 individuals) had the SS genotype. Significant effects of the LL + LS genotypes were observed, when compared to the SS genotype, on the cortical volume in the right anterior midcingulate gyrus and left anterior midcingulate gyrus.	L Allele × Volume in the right anterior midcingulate gyrus = 0.001 * L Allele × Cortical volume = 0.001 *	SS
Kao et al. [27]	5HTT mRNA level as a potential biomarker of treatment response in patients with major depression in a clinical trial	Investigate whether the serotonin transporter mRNA level (5HTT or SERT or SLC6A4) may be used as a biomarker of treatment response in MDD patients treated with different antidepressants while controlling related factors.	2018	China	119	VNTR (Stin2) SNP (rs25531)	PCR, RFLP, RNA extraction, real-time	Among the research participants, the majority of MDD patients treated with duloxetine or paroxetine had the SS genotype (72.2% and 66.2%, respectively). The increase in the 5HTT mRNA level correlated with the response to treatment.	Polymorphism × Fluoxetine = 0.042 *	VNTR = SS rs 25531 = S’S’ (Lg/Lg + Lg/S + SS)
Schneider et al. [28]	Association of Serotonin Transporter Gene AluJb Methylation with Major Depression, Amygdala Responsiveness, 5HTTLPR/rs25531 Polymorphism, and Stress	Investigate whether AluJb methylation on the SLC6A4 promoter is associated with MDD, amygdala reactivity to emotional faces, 5HTTLPR/rs25531 polymorphism, and recent stress.	2018	Germany	122	SNP (rs25531)	Sequencing and Magnetic Resonance	Most of the research participants in the MDD group had the LaSa genotype (48 individuals). People with two alleles with inherent risk appear to have lower AluJb methylation compared to carriers of an allele without risk.	AluJb methylation × polymorphism = 0.003 *	La/Sa
Bansal et al. [29]	Serotonin Signaling Modulates the Effects of Familial Risk for Depression on Cortical Thickness	Assess whether the effects of family risk were modulated by the polymorphic action linked to the serotonin transporter region (5HTTLPR).	2017	United States	129	VNTR	Magnetic Resonance and PCR	The S allele frequency was higher in the high-risk of MDD developing group (40.8%) than in the low-risk group (34.9%). The 5HTTLPR polymorphism modulated the familial risk effects of depression on the cortex, probably by modulating brain plasticity.	S Allele = 0.3390	LS
Schneck et al. [30]	Relationship of the serotonin transporter gene promoter polymorphism (5HTTLPR) genotype and serotonin transporter binding to neural processing of negative emotional stimuli	Examine the relationship between the 5HTTLPR genotype and the in vivo 5HTT binding, quantified by PET, with the amygdala reactivity to negative emotional stimulation.	2017	United States	21	SNP (rs25531)	Genotyping per Parsey et al. [31], functional magnetic resonance imaging, and positron emission computed tomography (PET)	Among the research participants, 10 had the LS genotype. The S allele presence did not correlate with the current severity of depression nor the number of depressive episodes throughout life. As for the amygdala reactivity, the 5HTTLPR gene was not associated.	S allele × Depression severity = 0.72 S allele × Depressive episodes = 0.09	L’S’
Kostić et al. [32]	A pilot study on predictors of brainstem raphe abnormality in patients with major depressive disorder	Analyze the possible association of brainstem raphe abnormal echogenicity in MDD patients compared to healthy individuals, and evaluate MDD clinical and genetic correlates.	2017	Serbia	53	SNP (rs25531)	PCR, RFLP, and transcranial ultrasound	There was no statistical difference between depressed participants with abnormalities in the brainstem’s raphe nucleus compared to the depressed group without abnormalities. However, the short allele (S) homozygote prevalence was significantly higher in depressed patients with abnormalities in the raphe nucleus. The LS genotype was the most prevalent in general.	Polymorphism × with or without raphe abnormality = 0.048 *	SL
Talati et al. [33]	Associations between serotonin transporter and behavioral traits and diagnoses related to anxiety	Examine the correlation between the 5HTTLPR variant and the anxiety and depression associated behavioral characteristics, verify this association with the clinical diagnosis, and explore whether the behavioral characteristics mediate the association between 5HTTLPR and anxiety/MDD.	2017	United States	203	SNP (rs25531)	PCR	The majority of the participants with at least some disorder had the SL genotype (116 participants, 57%). The same occurred in MDD participants (60 participants). In high-risk participants, the 5HTTLPR variant was associated with panic disorder and phobias.	Polymorphism× impulsivity = 0.0013 * Polymorphism × hostility = 0.017 * Polymorphism × neuroticism = 0.013*	SL
Jaworska et al. [34]	The influence of 5HTTLPR and Val66Met polymorphisms on cortical thickness and volume in limbic and paralimbic regions in depression: a preliminary study	Evaluate the influence of 5HTTLPR and Val66Met polymorphisms on cortical thickness in the cingulate, frontal, and parahippocampal regions, and the insula (areas modulated more consistently by these polymorphisms).	2016	Canada	43	SNP (rs25531)	Magnetic Resonance and PCR	Most MDD participants were S/La heterozygous (20 participants). In the MDD group, more significant volumes in the left thalamus and putamen were observed in the LA/LA homozygotes.	Polymorphism × Volume in putamen = 0.004 * Polymorphism × Volume in the thalamus = 0.005 *	S/La
Sun et al. [35]	Effects of polymorphisms of serotonin transporter promoter (5HTTLPR) and brain derived neurotrophic factor gene (G196A rs6265) on the risk of major depressive disorder in the Chinese Han population	Explore the 5HTTLPR and BDNF genes (rs6265) polymorphism and their possible interaction with the risk of MDD.	2016	China	459	VNTR	PCR	The majority of participants had the LL 5HTTLPR genotype, 232 in the case group and 231 in the control group. The LS heterozygous genotype promotes a significantly higher risk of developing MDD than the SS or LL homozygous genotype.	LS genotype × Depression = 0.02 *	SS
Manoharan et al. [36]	Serotonin transporter gene (SLC6A4) polymorphisms are associated with response to fluoxetine in south Indian major depressive disorder patients	Investigate the influence of the 5HTTLPR and rs25531 gene variants in response to fluoxetine treatment.	2016	India	126	VNTR SNP (rs25531)	Genotyping according to Kaiser et al. [37], and PCR real time	Most of the 5HTTLPR participants had the LS genotype, while in the rs25531 had the La genotype. The LL genotype and the LaLa haplotype of *SLC6A4* are associated with favorable treatment response to fluoxetine in MDD patients.	5HTTLPR × fluoxetine = 0.0066 * rs25531 × fluoxetine = 0.0818	VNTR = LS rs25531 = S/La
Ramasubb et al. [38]	Amygdala responses to quetiapine XR and citalopram treatment in major depression: the role of 5HTTLPR-S/Lg polymorphisms.	Examine the impact of two antidepressants with differential actions on the serotonin transporter and the 5HHTLPR polymorphisms on tonsil responses in MDD.	2016	Canada	57	SNP (rs25531)	PCR and RFLP	The La/Sa genotype was the most frequent in the groups. Citalopram did not affect amygdala responses in MDD patients with S or Lg alleles at weeks 1 and 8 compared to baseline. In contrast, Quetiapine decreased amygdala responses in MDD patients with S or Lg alleles, and changes in amygdala responses at week 8 correlated with a reduction in depression scores. The effectiveness of both treatments was comparable.	Medications in S/Lg genotype × Score HAM-A = 0.07 * Drugs in S/Lg genotype × Score HDRS = 0.11	La/Sa
Tatham et al. [39]	The 5HTTLPR and BDNF polymorphisms moderate the association between uncinate fasciculus connectivity and antidepressants treatment response in major depression	Assess whether the white matter integrity indices linked to 5HTTLPR serotonin transport and Val66Met brain-derived neurotrophic factor (BDNF) polymorphisms predict the magnitude of change in depressive symptoms after treatment with antidepressants.	2016	Canada	46	SNP	Magnetic Resonance, PCR, and RFLP	Most of the MDD participants (45.95%) had the SL genotype. When evaluating the effect of medication and the 5HTTLPR genotype, it was noted that most patients with remission had SL genotype. Moreover, the combined white matter integrity measures and genetic factors may help predict improvements in depressive symptoms after antidepressant treatment.	Treatment × Polymorphism = 0.55	L’S (La/Lg or La/Sa)
Tatham et al. [40]	White matter integrity in major depressive disorder: Implications of childhood trauma, 5HTTLPR and BDNF polymorphisms	Evaluate the influence of childhood trauma, 5HTTLPR and BDNF polymorphisms on myelin integrity in the brain of MDD patients.	2016	Canada	55	SNP	PCR, RFLP, and imaging exams	Most participants in the MDD group had the heterozygous SL genotype (49%). The SS genotype was not associated with reports of severe trauma when compared to the LL genotype. The results suggest that the frontal and limbic regions were affected by depression and influenced by childhood traumatic experiences and genetic risk factors.	Polymorphism × Trauma = 0.574	‘L’S (La/Lg or La/Sa)

* *p* < 0.05; VNTR = 5HTTLPR-VNTR; SNP = is not quoted by RefSeq; MDD = Major Depressive Disorder.

## Supplementary Materials

The following are available online at https://www.mdpi.com/2073-4425/11/11/1260/s1, Table S1: Checklist of GRIPS Statement on articles.
