# Supplementary material for: 5HTTLPR Genetic Variant and Major Depressive Disorder: A Review
Source: Genes (Basel). 2020 Oct 26;11(11):1260. doi: 10.3390/genes11111260 (PMC7692865; doi:10.3390/genes11111260)
Supplement: Supplementary file 1 [file genes-11-01260-s001.pdf]

Table 2: Check -list of GRIPS Statement on articles**.**

GRIPS Statement: Reporting recommendations for evaluations of risk prediction models that include genetic variants.																				
	**Recommendation**	**Basu et al. [12]**	**Camarena et al. [13]**	**Mendonça et al. [14]**	**Sarmiento Hernández et al. [15]**	**Ozçurumez et al. [16]**	**Fleurkens et al. [17]**	**Han et al. [18]**	**Kao et al. [22]**	**Schneider et al. [23]**	**Bansal et al. [24]**	**Schneck et al. [25]**	**Kostic et al. [27]**	**Talati et al. [28]**	**Jaworska et al. [29]**	**Sun et al. [30]**	**Manoharan et al. [31]**	**Ramasubb et al. [33]**	**Tatham et al. [34]**	**Tatham et al. [35]**
**Methods**																				
**Study design**	Present key elements of study design early in the paper	X						X	X	X	X	X	X	X	X	X	X	X	X	X
**Setting**	Describe the setting, locations, and relevant dates, including periods of recruitment, exposure, follow-up, and data collection				X	X	X	X	X	X	X	X	X	X	X	X		X	X	X
**Participants**	Describe eligibility criteria for participants, and sources and methods of selection of participants.	X	X	X	X	X	X	X	X	X	X	X	X	X	X	X	X	X	X	X
**Variables: Definition**	Clearly define all participant characteristics, risk factors and outcomes. Clearly define genetic variants using a widely used nomenclature system.	X	X	X	X	X	X						X	X	X	X	X	X	X	X

	Recommendation	Basu et al. [12]	Camarena et al. [13]	Mendonça et al. [14]	Sarmiento Hernández et al. [15]	Ozçurumez et al. [16]	Fleurkens et al. [17]	Han et al. [18]	Kao et al. [22]	Schneider et al. [23]	Bansal et al. [24]	Schneck et al. [25]	Kostic et al. [27]	Talati et al. [28]	Jaworska et al. [29]	Sun et al. [30]	Manoharan et al. [31]	Ramasubb et al. [33]	Tatham et al. [34]	Tatham et al. [35]
**Variables: Assessment**	(a) Describe sources of data and details of methods of assessment (measurement) for each variable.	X	X	X			X	X	X	X	X	X	X	X	X	X	X	X	X	X
	(b) Give a detailed description of genotyping and other laboratory methods.	X	X	X			X	X	X	X	X	X	X	X	X	X	X	X	X	X
**Bias**	Describe any efforts to address potential sources of bias	X				X	X							X		X				
**Study size**	Explain how the study size was arrived at		X								X			X		X				
**Analysis: Statistical methods**	Specify all measures used for the evaluation of the risk model including, but not limited to, measures of model fit and predictive ability.	X				X	X	X	X	X	X		X	X	X	X	X	X	X	X

	Recommendation	Basu et al. [12]	Camarena et al. [13]	Mendonça et al. [14]	Sarmiento Hernández et al. [15]	Ozçurumez et al. [16]	Fleurkens et al. [17]	Han et al. [18]	Kao et al. [22]	Schneider et al. [23]	Bansal et al. [24]	Schneck et al. [25]	Kostic et al. [27]	Talati et al. [28]	Jaworska et al. [29]	Sun et al. [30]	Manoharan et al. [31]	Ramasubb et al. [33]	Tatham et al. [34]	Tatham et al. [35]
**Other analyses**	Describe any methods used to examine subgroups and interactions	X	X				X	X	X	X	X		X	X	X	X	X		X	X
**Analysis: Missing data**	Specify how missing data were handled.								X	X			X							
**Other analyses**	If applicable, describe analytical methods taking account of sampling strategy																			
**Other analyses**	Describe any sensitivity analyses						X	X	X	X	X	X	X							
**Results**																				
**Participants**	(a) Report the numbers of individuals at each stage of the study. Give reasons for nonparticipation at each stage. Report the number of participants not genotyped, and reasons why they were not genotyped.	X	X	X	X		X				X		X				X	X	X	X

	Recommendation	Basu et al. [12]	Camarena et al. [13]	Mendonça et al. [14]	Sarmiento Hernández et al. [15]	Ozçurumez et al. [16]	Fleurkens et al. [17]	Han et al. [18]	Kao et al. [22]	Schneider et al. [23]	Bansal et al. [24]	Schneck et al. [25]	Kostic et al. [27]	Talati et al. [28]	Jaworska et al. [29]	Sun et al. [30]	Manoharan et al. [31]	Ramasubb et al. [33]	Tatham et al. [34]	Tatham et al. [35]
**Participants**	(b) Give reasons for non-participation at each stage		X										X				X		X	X
**Descriptives: Population**	(a) Report demographic and clinical characteristics of the study population, including risk factors used in the risk modeling.	X	X	X	X	X	X	X	X		X	X	X	X	X	X	X	X	X	X
	(b) Indicate number of participants with missig data for each variables of interest		X						X	X										X
**Outcome data**	Report numbers of outcome events or summary measures	X	X	X			X	X	X	X	X		X	X	X	X	X	X	X	X
**Analysis: Validation**	Specify the procedure and data used for the validation of the risk model.	X	X	X		X	X	X	X	X	X		X	X		X	X	X		X
**Other analyses**	Present results of any subgroup, interaction, or exploratory analyses, whenever pertinent.						X	X	X	X	X	X	X	X	X	X	X	X	X	X

	Recommendation	Basu et al. [12]	Camarena et al. [13]	Mendonça et al. [14]	Sarmiento Hernández et al. [15]	Ozçurumez et al. [16]	Fleurkens et al. [17]	Han et al. [18]	Kao et al. [22]	Schneider et al. [23]	Bansal et al. [24]	Schneck et al. [25]	Kostic et al. [27]	Talati et al. [28]	Jaworska et al. [29]	Sun et al. [30]	Manoharan et al. [31]	Ramasubb et al. [33]	Tatham et al. [34]	Tatham et al. [35]
**Discussion**																				
**Limitations**	Discuss limitations and assumptions of the study, particularly those concerning study design, selection of participants, and measurements and analyses, and discuss their impact on the results of the study.	X	X		X	X	X	X	X	X	X	X	X	X	X	X		X	X	X
**Interpretation**	Give an overall interpretation of results considering objectives, limitations, multiplicity of analyses, results from similar studies, and other relevant evidence.	X	X		X	X	X	X		X	X	X	X	X	X	X	X	X	X	X
**Generalizability**	Discuss the generalizability and, if pertinent, the health care relevance of the study results.	X	X			X	X	X	X	X	X	X	X	X	X	X	X	X	X	X

X = Presence.
